# Snake venom and cerebrovascular events: insights and public health implications

**DOI:** 10.3389/fpubh.2025.1513453

**Published:** 2025-02-05

**Authors:** Jorge Vasconez-Gonzalez, María de Lourdes Noboa-Lasso, Esteban Ortiz-Prado

**Affiliations:** ^1^One Health Research Group, Faculty of Health Science, Universidad de Las Americas, Quito, Ecuador; ^2^Program in Occupational Safety and Health, The University of Porto, Porto, Portugal

**Keywords:** snakebite, venom-induced stroke, stroke, neurovascular complications, public health

## 1 Introduction

Snakebites are recognized as one of the 20 neglected tropical diseases and represent a major public health concern in tropical and subtropical regions of Africa, Asia, and Latin America. This issue is particularly acute in remote, underdeveloped, and politically marginalized areas, where access to healthcare and preventive measures is limited (1, 2). According to the World Health Organization, approximately 4.5–5.4 million snakebites occur globally each year, resulting in 2.7 million clinical cases and an estimated 81,000–138,000 fatalities (3). The most vulnerable populations include individuals engaged in agricultural, livestock, fishing, or hunting activities, those living in poorly constructed homes, children involved in labor, and people with limited access to education (3).

Snakebite envenomation can cause a broad spectrum of complications, ranging from localized to systemic effects. These, bleeding disorders, kidney failure, severe tissue destruction, skin infections, compartment syndrome, and serum sickness, among others (4, 5). In severe cases, amputations may be necessary due to extensive tissue damage.

Venomous snakebites are also associated with significant neurological complications, as the venom is a complex mixture of toxins and enzymes, including phospholipase A2, acetylcholinesterase, hyaluronidase, and metalloproteinases. Neurological complications include ophthalmoplegia, ptosis, paralysis of pharyngeal muscles, paralysis of the intercostal muscles and the diaphragm (6–8). Among the most severe neurological complications are cerebrovascular accidents, which can be of the ischemic type (9–15), or also of the hemorrhagic type (16–21). Although stroke is a rare complication of snakebites, it can lead to death or serious sequelae. Little has been said about this complication; most reports come from clinical cases, and there is only limited information about its specific clinical characteristics, the assessment of its severity, and the duration of the follow-up period (22, 23).

In cases where a stroke occurs, whether ischemic or hemorrhagic, a variety of symptoms may appear. In cases of ischemic stroke caused by blockage of blood vessels, the most common symptoms reported include hemiparesis, altered speech (slurring of speech, dysphonia, dysarthria), drowsiness, ptosis, hypertension, and tachycardia (9–15). In cases of hemorrhagic stroke resulting from bleeding within the brain, the most common symptoms include headache, hypertension, hypotension, hematemesis, hematuria, bleeding gums, conjunctival hemorrhage, seizures, and respiratory distress. cardiorespiratory arrest, acute kidney failure and coma have also been reported (16–21). In both ischemic and hemorrhagic strokes, altered state of consciousness, loss of consciousness, miosis, altered pupillary reflex, and decreased muscle strength are present. The most commonly used diagnostic tests include computed tomography and magnetic resonance imaging, which have revealed that strokes affect medium to large vessels, including the middle cerebral artery and anterior cerebral artery (6, 24).

The present article aims to describe the characteristics of this type of complication, as well as the implications and public health interventions needed to address this problem.

## 2 Diverse components and biological effects of snake venom

The composition of snake venom is unique to each species and consists of a heterogeneous mixture of proteins and peptides (Table 1). The four dominant protein families in its composition are phospholipase A2, snake venom metalloprotease, three-finger toxins, and snake venom serine protease; these, in turn, are the most important toxins in human envenomation, responsible for causing coagulopathy, neurotoxicity, myotoxicity, and cytotoxicity (25).

**Table 1:** Specific components of snake venom and their effect on the organism.

**Specific venom components**	**Effect**
Phospholipases A2	Induced various biological effects such as neurotoxic, myotoxic, cytolytic, edematic, cardiotoxic and anticoagulant effects, inflammation, hypotension (45).
Fibrinolytic enzymes	Fibrinolytic activity, inhibition of platelet aggregation (45).
Metalloproteinases	Induce hemorrhage, proteolytic degradation of fibrinogen and fibrin, induction of apoptosis and inhibition of platelet aggregation (46).
Snake venom serine-proteases	Hypotensive effect through its kallikrein-like activity decreases blood fibrinogen levels, degrades angiotensin I and releases bradykinin from plasma kininogen with potent vasodilator effect (47).
Vascular endothelial Growth Factor Like (VEGF-like) Peptides	Possible hypotensive effect, due to VEGF-like mechanism of action (47).
Natriuretic peptides	Vasorelaxation, hypotension (45).
Bradykinin-potentiating peptides	Hypotension (45).
Disintegrins	Inhibition of platelet aggregation (45).
Three-finger toxins (3FTXs)	Hypotension, vasorelaxation, inhibition of platelet aggregation (45).
Nucleotidases	Hypotensive activity through vasodilation (47).

The table describes the specific effects on the organism of various components of snake venom.

Other venom components, such as sapharotoxins and endothelins, induce vasoconstriction of coronary arteries, while certain peptides inhibit angiotensin-converting enzyme (ACE), leading to a reduction in blood pressure (26). Aminopeptidases, on the other hand, contribute to systemic hypotension by altering vascular tone and fluid balance. Meanwhile, toxins that interfere with blood coagulation can cause hemorrhages or thrombosis, depending on the specific biochemical profile of the venom. Non-enzymatic proteins, such as C-type lectins and three-finger toxins, have also been identified. These proteins exhibit anticoagulant or procoagulant activity, functioning as agonists or antagonists of platelet aggregation, further increasing the risk of vascular complications (27–31).

## 3 Pathophysiological mechanism of cerebrovascular accident development after snake bite

Snake venom is a complex mixture of toxins and enzymes that can cause severe systemic and neurological effects. Among its components are neurotoxic, hemotoxic, and myotoxic substances, which can significantly disrupt the blood vascular system and alter microvascular dynamics, ultimately leading to strokes through various mechanisms following envenomation (26, 30, 32).

**Figure 1:**
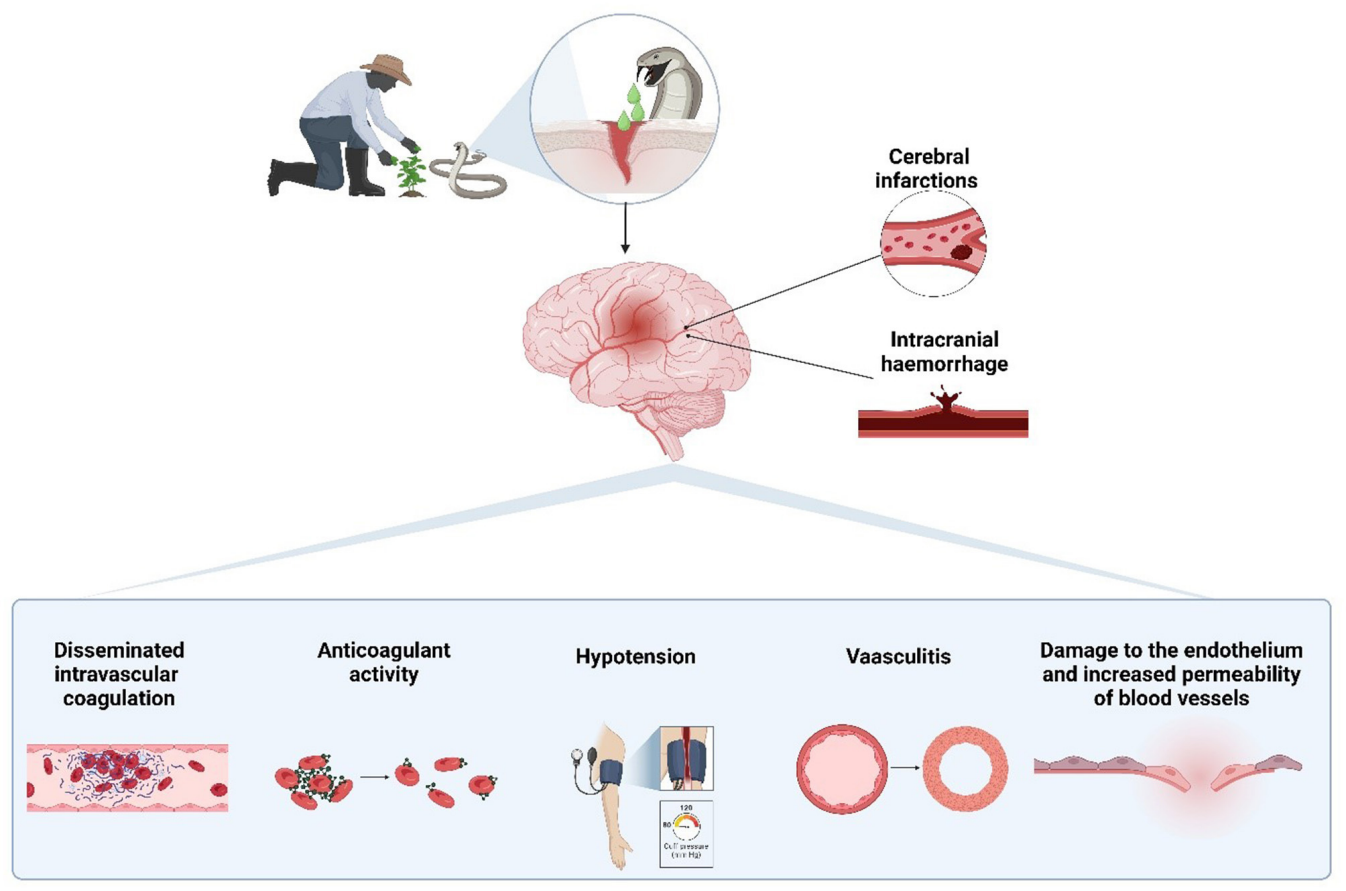
Pathophysiological mechanisms of cerebrovascular events induced by snakebite envenomation. Snake venom can lead to both cerebral infarctions and intracranial hemorrhages through various mechanisms, including disseminated intravascular coagulation, anticoagulant activity, hypotension, vasculitis, and endothelial damage, which increase vascular permeability. Created with BioRender.com.

The pathophysiology of cerebrovascular events associated with snakebites is multifactorial, involving processes such as thrombus formation, hypotension, and hemorrhage (Figure 1). Each of these mechanisms contributes to the risk of developing ischemic or hemorrhagic strokes. A comprehensive understanding of these processes is crucial for designing targeted interventions and improving clinical outcomes for affected individuals.

High concentrations of venom can induce disseminated intravascular coagulation (DIC), leading to the formation of thrombi that can block blood vessels. Additionally, direct damage to the vascular endothelium by venom can cause vasculitis, resulting in thrombosis. Venom's cardiotoxic effects may also promote cardiac thromboembolism, while hyperviscosity due to hypovolemia and hypoperfusion exacerbates the risk of vascular occlusion (33, 34). All these processes lead to the formation of thrombi which can cause an ischemic stroke. For its part phospholipase A2 isoforms present in venom can cause significant vasodilation, leading to hypovolemia from sweating, vomiting, and reduced fluid intake. This can result in hypotension, which in turn may cause watershed infarction, a type of stroke occurring in the regions of the brain that are most susceptible to low blood flow (6, 34).

Hemorrhagins, another venom component, damage endothelial cells and increase blood vessel permeability, potentially leading to spontaneous intracerebral bleeding. The risk of hemorrhage is further heightened by venom-induced thrombocytopenia, as well as the prolongation of prothrombin and partial thromboplastin times, which impair the blood's ability to clot and favor bleeding (4, 35, 36).

## 4 Coagulopathy and its role in bleeding and vascular rupture

Snake venom-induced coagulopathy is a critical factor contributing to severe bleeding and vascular complications following envenomation. Certain venom components, such as metalloproteinases and C-type lectins, disrupt normal coagulation pathways by interfering with platelet aggregation and degrading fibrinogen, a key protein in blood clot formation (29). This dysregulation can lead to disseminated intravascular coagulation (DIC), characterized by widespread clot formation that depletes clotting factors and platelets, ultimately resulting in a paradoxical increase in bleeding (33, 34). Additionally, venom-induced thrombocytopenia further reduces the blood's clotting ability, while the degradation of endothelial integrity by hemorrhagins weakens blood vessels. These combined effects can culminate in vascular rupture and spontaneous intracerebral bleeding, posing life-threatening risks to affected individuals. Understanding the mechanisms underlying coagulopathy is essential for developing therapeutic strategies to mitigate these complications (37).

## 5 Epidemiology of snakebite-related strokes

Strokes are a serious but often underreported complication of snakebites. The true incidence of stroke following snakebites remains largely unknown due to significant underreporting and limitations in existing data. Most available information comes from case reports and small case series, making it difficult to accurately assess the true burden of this complication. A 1991 study in Ecuador found that 5.1% of 294 patients who suffered snakebites developed intracranial hemorrhages, although this study was limited by its reliance on clinical diagnosis without imaging studies (38). A more recent Ecuadorian study from 2003 reported that 2.6% of 309 patients bitten by *Bothrops* snakes developed cerebrovascular events, with CT scans confirming the presence of intracranial hemorrhages and cerebral infarcts (35). In Sri Lanka, a 2007 case series involving 500 patients bitten by Daboia russelii found that nine developed ischemic strokes (6). A scoping review further highlighted that among stroke cases following snakebite, 77.1% were ischemic strokes, 20.5% were intracranial hemorrhages, and 2.4% were infarct-like hemorrhages (24).

Data from the last 5 years (2019–2024) reveals 23 reported cases of stroke caused by snakebites, including 12 hemorrhagic strokes and 11 ischemic strokes. Most of these cases occurred in the male population, with the majority caused by snakes of the genus *Bothrops* (Table 2). The lack of studies on this topic is a major problem, as the absence of data makes it difficult to determine the magnitude of the issue, identify the most affected areas, and develop programs or strategies for its control. For this reason, it is crucial to emphasize the importance of conducting large-scale studies to obtain more robust data on all the regions with a high incidence of snake bites.

**Table 2:** Cases of stroke caused by snake bites during 2019–2024.

**Research**	**Snake**	**Stroke type**
Ansoumane Hawa et al. (17)	NA	Hemorrhagic
Dabilgou et al. (18)	NA	Hemorrhagic
Dabilgou et al. (18)	NA	Hemorrhagic
Dabilgou et al. (18)	NA	Hemorrhagic
Ladgani et al. (19)	NA	Hemorrhagic
Nascimento et al. (16)	*Bothrops atrox*	Hemorrhagic
Pérez-Gómez et al. (20)	*Bothrops atrox*	Hemorrhagic
Pérez-Gómez et al. (20)	*Bothrops atrox*	Hemorrhagic
Pérez-Gómez et al. (20)	*Bothrops atrox*	Hemorrhagic
Sachett et al. (21).	*Bothrops atrox*	Hemorrhagic
Sachett et al. (21).	*Bothrops atrox*	Hemorrhagic
Senthilkumaran et al. (48)	*Daboia russelii*	Hemorrhagic
Sirur et al. (14)	*Hypnale*	Hemorrhagic
Bentes et al. (39)	*Bothrops atrox*	Ischemic
Ghosh et al. (9)	*Daboia russelii*	Ischemic
Lahiri et al. (10)	NA	Ischemic
Martínez-Villota et al. (11)	*Bothrops*	Ischemic
Namal Rathnayaka et al. (12)	*Hypnale spp*	Ischemic
Pinzon et al. (13)	*Calloselasma rhodostoma*	Ischemic
Senthilkumaran et al. (49)	*Bungarus caeruleus*	Ischemic
Senthilkumaran et al. (49)	*Daboia russelii*	Ischemic
Senthilkumaran et al. (49)	Cobra (Species not mentioned)	Ischemic
Sirur et al. (14)	*Hypnale*	Ischemic
Smith and Brown (50)	*Pseudonaja textilis*	Ischemic
Srinath et al. (51)	*Daboia russelii*	Ischemic
Sun et al. (15)	*Protobothrops mucrosquamatus*	Ischemic
Zeng et al. (52)	*Trimeresurus stejnegeri*	Ischemic

The table summarizes the types of stroke (ischemic and hemorrhagic) and the snakes involved.

## 6 Risk factors for the development of stroke after snake bite

There is no conclusive information on the risk factors that predispose individuals to the development of stroke after sepsis; a case series conducted in 2019, in which 83 cases were reported, mentions that the majority of cases involved patients under 50 years of age who did not present comorbidities or risk factors for hemorrhagic or ischemic stroke. Only 2% of patients had a history of underlying comorbidities that could be potential risk factors, such as diabetes mellitus or hypertension (24). Another case series indicates that the appearance of confusion, mild reduction in the Glasgow coma scale, or hemiparesis should be followed by additional investigation with brain imaging (6).

Regarding the type of snakes, ischemic strokes are more frequent after envenomation by snakes of the *Viperidae* family. Despite this, it has been observed that *Bothrops* snakes are more prone to developing hemorrhagic strokes; however, it is important to mention that this also depends on the specific species of snake. In cases of *Bothrops atrox* envenomation, up to 12.8% report hemorrhagic cerebrovascular alterations. *Bothrops lanceolatus* and *Bothrops caribbaeus* are typically associated with cases of ischemic strokes (39). Likewise, poisoning by *Daboia russelii* snakes is found to be related to the development of ischemic strokes (6).

## 7 Public health implications and interventions for snakebite-related complications

The public health implications of snakebites extend far beyond the immediate medical consequences. While well-known complications such as amputations are concerning, cerebrovascular events (such as strokes) induced by snake venom can result in long-term disabilities with severe health and socioeconomic repercussions. Stroke, in particular, leaves patients with debilitating sequelae that increase their dependence on the healthcare system. Snakebite victims are often located in remote areas with limited access to healthcare, which exacerbates the challenges of providing timely treatment and rehabilitation. This geographical isolation leads to delayed treatment, resulting in poorer prognoses. Patients with a poor prognosis will likely have diminished ability to work, reduced capacity to care for their families, and increased reliance on medical treatments such as pharmaceuticals, further inflating healthcare costs.

Given the substantial burden that snakebite complications impose on affected individuals and communities, targeted public health interventions are crucial:

**- Enhance surveillance systems:** Improve the reporting infrastructure to gather detailed data, including the species involved and the location of incidents, ensuring consistency and better preparedness in public health responses (40).

**- Implement preventive campaigns:** Launch education initiatives in high-risk areas, emphasizing preventive measures like using flashlights at night, avoiding walking barefoot, and wearing protective clothing while engaging in agricultural work. These campaigns should also focus on reducing snake habitats near homes by eliminating rodents and clutter (30).

**- Educational outreach in rural areas:** Educate communities, particularly in rural and remote areas, on the benefits of prompt medical intervention post-snakebite. In many regions, reliance on traditional medicine due to cultural beliefs or lack of healthcare access delays critical care, worsening outcomes. Educational outreach can help bridge this gap and reduce the time to treatment (41–43).

**- Improve access to antivenom and medicines:** Address the lack of access to essential treatments, particularly antivenoms. Ensuring a reliable supply of antivenoms is vital, as shortages, distribution inefficiencies, and dependence on imports exacerbate the health risks in regions where snakebites are prevalent. Efforts should focus on promoting local production of antivenom suited to regional snake species and improving the distribution system to reach remote areas (41, 44).

**- Evaluate healthcare capabilities:** Continuously assess the availability and effectiveness of healthcare services in treating snakebite complications, including stroke. This includes providing adequate diagnostic tools, replacement therapies, and mechanical ventilators. Training healthcare workers on the best practices for treating snakebite-related cerebrovascular events is crucial to improving outcomes (40, 41).

**- Public health investment:** Encourage investment from both government and private entities to fund the development of affordable and effective antivenoms that are widely accessible. This will help alleviate the financial burden on health systems and ensure that treatments are available in underserved regions (42).

**- Research and development:** Promote research aimed at understanding the pathophysiological mechanisms that lead to cerebrovascular events following snakebites. This research is critical for developing specific therapeutic interventions that can reduce the long-term impacts of snakebite-related strokes (24).

The combination of long-term health effects, such as reduced productivity and increased healthcare costs, makes snakebites not only a medical issue but a significant public health concern. Addressing the lack of resources, such as antivenoms, medicines, and medical infrastructure, particularly in remote areas, is essential for mitigating the widespread impacts of snakebites. By focusing on prevention, timely access to treatment, and ongoing research, we can significantly reduce the burden of snakebites on individuals and communities.

## 8 Conclusion

Snakebites represent not only a clinical emergency but also significant public and global health challenges, particularly in remote and underserved regions where access to healthcare is limited. Beyond the immediate physical complications, the long-term effects of cerebrovascular events, such as strokes, lead to severe disabilities, reduced economic productivity, and increased dependence on healthcare systems. These consequences underscore the need for integrated public health strategies, including improving healthcare infrastructure in rural areas, addressing shortages of essential medicines such as antivenoms, and raising public awareness through preventive campaigns. By addressing these issues from a public health perspective, the long-term socioeconomic and health impacts of snakebites can be mitigated, improving the quality of life for affected populations and reducing the global burden of neglected tropical diseases.

The reported incidence of snakebite-induced strokes in Sri Lanka, India, and Ecuador highlights the need for targeted public health interventions in these affected areas. It also emphasizes the necessity of epidemiological studies in regions with a high incidence of snakebites to better understand the occurrence of snakebite-induced strokes and identify risk factors contributing to their development. These interventions should focus on awareness, education, and management, including improved access to antivenoms and better strategies for stroke prevention and management. Finally, further research is essential to understand the specific mechanisms of snake venom-induced strokes and identify the venom components of the primary snake species involved in these events. This will help develop more effective antidotes and treatments tailored to each case.
